# Whitlow

**Published:** 1884-06

**Authors:** C. Carleton Smith

**Affiliations:** Philadelphia


### "WHITLOW."

C. Carleton Smith, M. D., Philadelphia.

A short time since a lady came into my office and handed me a little package, which, on being opened, revealed to my astonished gaze the first and second phalanges of the fore-finger of her left hand.

"This," she said, holding up the remaining stump of the member, "is the result of allopathic treatment of a felon, with which I have suffered perfect torment, not only from the painfulness of the disease itself, but also from six repeated lancings at the hands of my physician; and after all, I am maimed for life."

As I held these necrosed bones in my hand and listened to the sufferer's pitiful story, and beheld the mangled suppurating stump which was all that was left to her as a reward for her endurance of so great torture, which she so patiently suffered under promise of a cure by so-called scientific treatment, my mind wandered back to the past and recalled an article which bears directly on this subject, written by von Grauvogl in 1861.

These are his words, which I quote in full:

"If a physician of the physiological school is called to a patient, who shows him a finger with the extreme phalanx swollen all around, deeply reddened, very painful, on the roof of the nail already formation of pus, he will, as he supposes that each and every paranitium has to be put over the same last, without hesitation, run the lancet in and order poultices. This is all he knows. Now the suppuration spreads in spite of all, breaks through the joint, and appears on the inside of the finger. This gives him a chance to make a deep, long incision there also, out of which, finally, the phalangeal bone, in a necrotic state, has to be taken out.

"Homœopathy teaches differently. The homœopathician inquires about concomitant circumstances. Suppose, now, he finds the patient is looking sickly and pale; in the morning feels weary and dull in the head; complains of having no appetite; evening chilly and feverish; the pain in the finger is rather better out-doors than in-doors; and the physician, examining this, finds as a permanent cause very damp walls, or a damp cellar, what can the physiological doctor do but poultice and cut, in spite of frequently experienced unfavorable results?

"If, after a few days, the homœopathician finds a blister on another finger of the same patient, and if, on inquiry, he ascertains that this also has been the case with the now suffering finger, still the physiological doctor does not know what to do with all this anamnesis but to poultice and cut as soon as possible. But homœopathicians are led by these concomitant circumstances to compare the provings of Natr. sulph.; give it in homœopathic doses, and in a few days both fingers will be well, as I can confirm by my own practice. Neither poulticing nor cutting will help, and, as I have also experienced, neither Ledum., Ars., nor Silicea.

"There is a paranitium from external hurts, and there is one from the remaining consequences of other external causes in the interior organism. Among other remedies for the paranitium, we have the Ledum and Silicea; the use of both has shown that Silicea will cure the interior consequences of external causes and not those of external injury. Ledum has been proved to cure pure consequences of injuries, but only in the first stage.

"If grangrene has already set in Ars. will cure. In both, the so-called physiological school has possibly to wait for a spontaneous exarticulation, or else make an amputation. In the latter case they would not even be able to prevent the death of the patient."

Comparing the results of this barbarous treatment with those of Homœopathy, how benign is the method of *similia*, and how universally successful is the curative result when we follow the teachings of the master; and with what confidence can we promise to our patients, thus afflicted, a cure without resort to the knife, and without shedding a drop of blood.

Besides the remedies spoken of in the above article, there are others which we will here consider, and which in our experienec have, when given according to their indications, been found entirely reliable, bringing quick relief to many a sufferer, and eliciting also their admiration and their thanks, for a method of cure so mild, and which accomplishes its end with perfect freedom from after deformity.

These remedies which I have found so uniformly successful when properly used, and which seem to be frequently called for, are as follows: Apis., Nux, Hepar, Sepia, Dioscorea vil.

If an Apis case presents itself for treatment, we find these two symptoms always present: first, a *burning stinging* pain; second, the affected part is *hard* and has a *white, sickly, bleached-out* appearance characteristic of a bee-sting.

If a Nux case falls into our hands, we find these symptoms prominent, viz.: the suppuration is in the *palmar* surface of the finger or a thumb as the case may be, pain *throbbing*, with sometimes *burning*. The throbbing is always aggravated by *warmth*, and letting the member *hang down*, and also worse in the *evening* after sundown; amelioration in bed.

If we meet with a case requiring Hepar (that much abused drug) we will find a decided *yellow color* of the skin, and the pus can be plainly felt beneath it as it is displaced by gentle pressure; there will also be present *throbbing*, and the patient cannot bear the weight or pressure of a poultice.

If we have a Sepia case to contend with, these symptoms must present themselves as our guide: *itching* with *throbbing*, *shooting* and *burning* at intervals or alternately; the part is *dark-red* aud pus is visible.

A Silicea case always has *tearing pains* as if the bones would actually be torn out, preventing all sleep; and it is the first remedy to think of in patients who suffer from chronic fœtid foot-sweat, or who are continually raising crops of boils.

We must think of Dioscorea vil. if the whitlow is found in the middle finger of either hand with a sensation as of a *brier* imbedded in the palmar surface; with tenderness on pressure similar to Hepar, though not so marked, and sharp pain not only in the affected portion, but flying from one finger to another.

Ledum is very apt to be the proper drug if we find out that the paranitium has been caused by pulling off abruptly a hang-nail.

In old cases which have come to us through other hands badly maltreated and in a gangrenous condition, emitting an intolerable odor, Dr. von Grauvogl relies upon Arsen., but my experience has led me to place *Lachesis* first before Ars. and it will be found that scarcely any other remedy will be needed in such a condition.

While preparing this paper a very intelligent lady entered my office, seeking relief from a whitlow, which was forming very rapidly on the palmar surface of the first finger of the right hand, pus already beginning to form with intense throbbing, swelling, redness, and acute darting pains. My first question was, "How did your finger get into this condition?" The answer was, "Well, very foolishly, I tore off a hang-nail that has been giving me a good deal of trouble, and this condition of things immediately followed." This lady had a felon once before and she begged to be saved from going through so much suffering again if possible. I told her that it was more than possible, and advised her not to apply anything in the shape of a poultice to it, but simply suspend the hand in a sling and take the powders I prescribed for her. In less than forty-eight hours my patient reported, and exhibited the suffering member almost well, the throbbing heat and pus almost completely gone. The remedy was *Ledum*$^{200}$.

These, then, are some of the principal remedies with their characteristic indications grouped together for the benefit of the doubting Thomases in our ranks; which I ask them, in all honesty to faithfully test, as I have tested them time and again. For I can assure these brethren of little faith, that if they will but be true to Homœopathy, Homœopathy will be true to them.

